# Evaluation of calling algorithms for array-CGH

**DOI:** 10.3389/fgene.2013.00217

**Published:** 2013-10-25

**Authors:** Siddharth Roy, Alison Motsinger Reif

**Affiliations:** ^1^Department of Statistics, College of Physical and Mathematical Sciences, North Carolina State UniversityRaleigh, NC, USA; ^2^Bioinformatics Research Center, North Carolina State UniversityRaleigh, NC, USA; ^3^UNC Institute for Pharmacogenomics and Individualized Therapy, University of North Carolina at Chapel HillChapel Hill, NC, USA

**Keywords:** array CGH, change point model, methods comparison, copy number variation, scan statistics

## Abstract

Copy number variation (CNV) detection has become an integral part many of genetic studies and new technologies promise to revolutionize our ability to detect and link them to disease. However, recent studies highlight discrepancies in the genome wide CNV profile when measured by different technologies and even by the same technology. Furthermore, the change point algorithms used to call CNVs can have substantial disagreement on the same data set. We focus this article on comparative genomic hybridization (CGH) arrays because this platform lends itself well to accurate statistical modeling. We describe some newer methodological developments in local statistics that are well suited for CNV detection and calling on CGH arrays. Then we use both simulation studies and public data to compare these new local methods with the global methods that currently dominate literature. These results offer suggestions for choosing a particular method and provide insight to the lack of reproducibility that has been seen in the field so far.

## Introduction

The identification of copy number variations (CNV) has been integral in improving our understanding of the molecular basis for many diseases. A CNV region represents a deviance in copy number from a reference genome that will typically contain 2 copies of each DNA segment (Sebat et al., 2004; Zhang et al., 2009). The different copy number in the DNA can cause dramatic effects in the levels of mRNA and protein levels which can impact many cell processes and lead to diseases such as cancer (Curtis et al., 2012). Currently, CNV regions have been found to be useful markers for improving diagnostics, finding disease subtypes, understanding response to therapy, and even performing comparative studies between species (Diskin et al., 2009; Zhang et al., 2009; Thomas et al., 2011; Curtis et al., 2012).

CNV are measured through both array-based technologies and sequencing based technologies. While sequencing platforms hold promise for CNV detection, array based platforms are the primary technology used to identify CNVs useful for diagnostics. These platforms have developed rapidly to provide increased genome resolution that should provide increased power to detect smaller CNV. However, an alarming number of studies have reported discrepancies when comparing calls from a replicate sample measured on different platforms and even on the same platform (Baumbusch et al., 2008; Curtis et al., 2009; Pinto et al., 2011). Further complicating this is that many studies have shown that different algorithms will provide different calls on the same sample (Lai et al., 2005; Winchester et al., 2009; Pinto et al., 2011). It is common practice to focus on regions identified from two different methods and to remove all calls that are smaller than five probes (Pinto et al., 2011). However, it has been noted that many of the removed regions detected only by one method can be validated (Conrad et al., 2010; Pinto et al., 2011).

SNP arrays have quickly become the dominant platform for CNV detection in human studies due to a higher resolution of probes with CN measurements. They also allow for the inclusion of SNPs, reference genomes, and other sources of information to improve power (Scharpf et al., 2008). However, comparative genomic hybridization (CGH) arrays remain common amongst scientists who study model organism due to the lack of available resources or poor reference genome. CGH arrays have a simple mean shifts structure for which segmentation methods have been developed. These methods are relatively simple compared to the Hidden Markov Models (HMM) used for SNP arrays and they model the data more accurately (Scharpf et al., 2008). This allows for a better understanding of why these methods differ and which one to use.

The common methods for CGH arrays, Circular Binary Segmentation (CBS) (Olshen et al., 2004; Venkatraman and Olshen, 2006), ADM-2 (Agilent Technologies), Nexus (Nexus Copy Number), and Fused Lasso (Tibshirani and Wang, 2008) are all global methods meaning they compare potential CNV regions to the full genome. They can also effectively detect aberrations of any size. This also allows them to be used for cancer studies for which large aberrations are typical, but it implies these methods may not fully take advantage of the sparse structure present in normal genomes. Recently, two new local segmentation algorithms have been proposed that focus on detection of CNV from high-resolution data sets (Jeng et al., 2010; Niu and Zhang, 2012). Not only are these methods a major conceptual change from the popular global change point methods, they have strong theoretical justifications which may guide intuition on detection limits. This improved understanding of the detection limit may provide insight into the lack of concordance between methods.

In the current paper, we will review current global techniques and then contrast them with these new methods. Next we will perform power analysis to understand the benefits and drawbacks of local vs global inference methods and to provide guidance to investigators considering different approaches. Finally, we will evaluate all methods on publically available data to evaluate and understand the concordance between methods. Results indicate that at least for the array CGH case, the problem is clearer for why one method may work better than another.

## Materials and methods

### Global segmentation methods

The most popular methods in statistics to detect multiple unknown change points are recursive binary segmentation methods (Killick et al., 2012). CBS (Olshen et al., 2004; Venkatraman and Olshen, 2006), ADM-2 (Agilent Technologies), and Nexus (Nexus Copy Number) are all based of this simple and yet powerful and effective procedure. These methods simplify the problem of finding multiple change points by searching for them one at a time. This is equivalent to performing forward selection. Each procedure starts by finding the most likely 1 or 2 change point locations on the chromosome. This is determined by defining a test statistic (usually a *t*-test) comparing the probe averages between the proposed change point locations, and the probe averages outside this window. Once the locations are found, a significance criterion is evaluated. If it is met, then the chromosome is split into 2–3 segments and the procedure is repeated on each newly formed segment. The procedure stops when significance is no longer met.

The advantage of these methods is that they are typically fast enough for modern data sets and they are easy to implement given a significance criterion. However, determining the correct cut off is not trivial (Olshen et al., 2004). Also, compared to other methods such as the Fused Lasso (Tibshirani and Wang, 2008), these methods are difficult to extend in a simple and fast way to include multiple sources of information such as B allele frequencies.

Penalized regression methods have also been popular for addressing the CNV problem and researches have found much more success generalizing them to larger models (Zhang et al., 2010). Each of these methods minimizes an objective function that consists of the sum of squares of the residuals plus some penalty terms that promote scarcity in calls and break points. The most common method is the Fused Lasso (Tibshirani and Wang, 2008) that uses an L1 penalty for both the coefficients as well as the difference in neighboring coefficients.

The major benefit to penalized regression methods compared to binary segmentation is that it is minimizing an objective function that should result in a global minimum. However, the major drawback in that one must choose tuning parameters and this can dramatically affect the answer.

#### CBS

CBS searches for change points 2 at a time and searchers for the maximum *t*-test statistic comparing the averages of the probes between the proposed change point locations to the averages outside of the proposed change point locations. It determines significance by using permutation tests by rearranging the probes. The permutations implemented are an approximation that allows CBS to scale well. Each segment is tested independently of other segments and this allows CBS to find very small regions amongst large regions that can commonly be seen in cancer genomics.

Using *p*-values as a stopping criterion in a forward selection type method is generally considered bad practice (Zhang and Siegmund, 2007). They lose their interpretation when number of change points is unknown in advance essentially due to the large amounts of multiple testing (Olshen et al., 2004; Zhang and Siegmund, 2007). An mBIC procedure had been developed and this is more consistent with current statistical practice. However, this procedure can tend to be over conservative and remove CNV that have been validated (Zhang and Siegmund, 2007). Both *p*-value and mBIC versions are easy to use and we will compare both in simulations.

#### ADM-2

This method is provided by Agilent technologies and it finds the change point that maximizes the *t*-test of comparing the averages between change points to 0 (Agilent Technologies). When a segment is kept, it is median centered and the procedure is repeated on the three new segments. This effectively combines the segmentation and calling process into one step.

The main drawback to this algorithm is that the significance threshold for the *t*-test values is a set user defined threshold. This makes it less automated and more subjective than CBS. However, tuning the value allows for an easy and intuitive way of dealing with large amounts of confounding that is present CNV studies. It is also substantially faster than using permutation tests. ADM-2 also uses Agilent computed standard errors to weight log ratios and reduce the effects of bad probes. This can be useful if done accurately.

#### Nexus

Nexus employs a ranking procedure prior to segmentation (Nexus Copy Number). Ranking is typically used to reduce the effects of extreme outliers. While, outliers do tend to exist, it is well accepted that most of the log ratio probes can be well approximated by a normal or slightly heavy tailed symmetric distribution. This implies that the Nexus procedure may be dramatically throwing away power.

After ranking, Nexus uses the same mean shifts testing procedure as CBS except it uses a normal distribution to determine significance to speed computation. However, using a normal distribution is a very inappropriate way of approximating the null distribution for maximum *t*-test type statistics (Olshen et al., 2004). If ranking were not employed, this would result in a large numbers of false positives.

### Fused lasso

The Fused Lasso method as originally proposed (Tibshirani and Wang, 2008) minimizes the following criteria

$$\begin{array}{l} \hat{\beta }=\arg\min\sum_{i}{\left(y_{i}-\beta_{i}\right)}^{2}\\ \text{subject to}:\sum_{j}\left|\beta_{j}\right|\leq s_{1},\sum_{j}\left|\beta_{j}-\beta_{j\;+\;1}\right|\leq s_{2} \end{array}$$

The global solution found by the method is entirely dependent on the choice of tuning parameters. The suggestion in the original paper, which was developed for large copy number aberrations in cancer, is to use a smoothed estimate of the CNV profile to get a crude estimate of the bound for both penalties. This tended to give a slightly smoothed but useful estimate of the cancer profile. More modern implementations suggest starting at the null flat solution and then to gradually increase the tuning parameters (Zhang et al., 2010). Each additional change point or region that is formed is penalized by BIC. This finds a minimum that is the estimated CNV profile.

### Local segmentation methods

While global segmentation compares the mean differences between regions across the genome, the newer local methods scan the genome to find the most probably change points or CNV. SaRa (Niu and Zhang, 2012) and LRS (Jeng et al., 2010) have emerged as promising new approaches for calling/detecting CNVs. Both elegantly show that the power of detection of a change point or a region is directly proportional to

$$T=\frac{n\left(\mu /\sigma^{2}\right)}{\log N}$$

where *N* is the total number of the probes on the chromosome, *n* is the number of probes in a CNV, μ/σ is the signal to noise ratio of the average of probe log ratios in the segment to overall noise on the array. In other words, the test statistic that determines power for testing the change point or region is proportional to a *t*-test divided by the log_e_ of the total number of probes.

#### SaRa

This new procedure introduces a novel sliding window approach to find probes with a high probability of being a break point (Niu and Zhang, 2012). After screening a list of high probability probes, this procedure uses backwards selection to find a final change point configuration. The advantage here is that the approach is intuitive and unlike binary segmentation, it can be theoretically shown to have a high probability of detecting all breakpoints if the correct window size is used. However, as with any sliding window approach, it is a challenge to choose an accurate window size. The author's recommend using multiple window sizes to form a pool of potential change points. The current recommendation is to use 3 window sizes corresponding to 1, 2, and 3 times the log_e_ of the total number of probes. These are then pruned with backwards selection using mBIC as described above.

#### LRS

The final method is appropriate for use only for germ line CNV data (Jeng et al., 2010). Similar to ADM-2, it combines calling and break point detection by identifying regions that are significantly different from 0. The first step is to scan the genome for any aberrations surpassing an extreme value threshold with width less than a pre chosen length L. The located regions are then summarized into non-overlapping CNV calls. By reducing the size of the alternative distribution of regions to be constrained within regions of length L, this method can be theoretically shown to be having high power to find all regions that surpass the given threshold.

The main assumption for this algorithm is that L is specified to be larger than the width of all present CNV but smaller than the distance between any two CNV. One could use previous experience to choose L [i.e., 100 probes is a reasonable setting (Jeng et al., 2010)] or a second algorithm could be used to justify or tune the parameter adaptively. A sensitivity analysis could also be performed to focus on regions that are called differently for various choices.

### Simulation set up

Our goal in this paper is to compare the ability of these global and local methods to detect CNV using standard implementations. Thus, we will borrow a simple but effective simulation set up from the local change point papers (Jeng et al., 2010; Niu and Zhang, 2012). The factors we vary are

1. *N*: total number of probes will vary between 5000, 10,000, and 20,000. This is the typical range of probes per chromosome seen on the Agilent 244 K data set that we evaluate in the real data analysis.
2. For each value of *N*, the length of the segment, *n*, varies from log_e_(*N*) to 5 log_e_(*N*).
3. The signal to noise of the segment μ/σ is varied from 0.8 to 3
4. The measurement error noise will be generated both from a normal distribution, which is the standard assumption, and from a heavy tailed distribution. We used a t distribution with 8 degrees of freedom for the heavy tailed distribution because it represents the measurement error seen in the real data below.

The segment width and signal to noise were chosen to represent a range of values from difficult to detect to easy to detect. This should provide better intuition for discrepancies in methods for real data. Five-hundred sample profile for each factor combination were evaluated. Each sample contains CNV of each width and these CNV are evenly spaced across the genome. We evaluate the methods described above across these different factors on their ability to detect aberrations and compare the number and pattern of false positive break points.

### Real data

Recently 6 HapMap samples (Pinto et al., 2011) were collected in triplicate on 11 of the common technologies used to date. The results from this study were that not only are the platforms qualitative different, but popular methods can give different answers as well on the same sample. We selected 3 HapMap samples and pulled data from the Agilent 244 CGH array to evaluate methods. The samples chosen were NA10851, NA18517, and NA12239. All samples in the study were normalized to NA10851 so we also evaluate the NA10851 self-self hybridizations because this set of technical replicates allows us to evaluate the array influence in causing false positives. There exist many methods for using self-self hybridizations to remove false positives for the rest of the samples in a study (Khojasteh et al., 2005; Lee et al., 2011) but there does not appear to be a consensus on which to choose. We choose to simply evaluate the patterns of false positives using standard implementation of the above methods, compare these patterns to results from simulations, and evaluate how well this can be used to improve concordance for other samples. The 3 technical replicates for each sample will allow us to evaluate how well each algorithm identifies reproducible CNV as well as what combination of algorithms provides the largest detection ability.

### Implementation

CBS is implemented using the DNAcopy package (Olshen et al., 2004) in R (R Core Team, 2013). No default settings were modified. The Fused Lasso implementation was performed using the cghFLasso package. We let the software choose the tuning parameters using the default smoothing technique. Since this method typically results in a smoothed estimate, we segmented the smoothed estimate by using a threshold at 0.5. This reduced the large number of break points that would be detected otherwise but still allowed us to observe whether the true break points were detected. Software to implement the SaRa and LRS algorithms was kindly provided by the authors of the methods. The main tuning parameter for LRS is the max width of the scan statistic (L). This was chosen to be so that the scan statistic would be larger than all segments used in all but the largest simulation. The threshold to keep a region was sqrt[2 * log(N * L)] where N is the total sequence length. We also used 3 window sizes for the SaRa procedure, which are proportional to 1, 2, and 3 times the log_e_ of the number of probes. This was recommended by the original paper and shown to perform well compared to the algorithm using and 1 window size alone. These window sizes completely coincide with the length of the aberrations we are trying to detect so it should maximize power for the sliding window. The global algorithms have a computational complexity of O(N^2) while the local algorithms have a complexity of O[Nlog(N)]. Thus, each of these methods are fast and can easily be run on large data sets efficiently on basic desktop or laptop machines.

## Results

### Power

**Figure 1:**
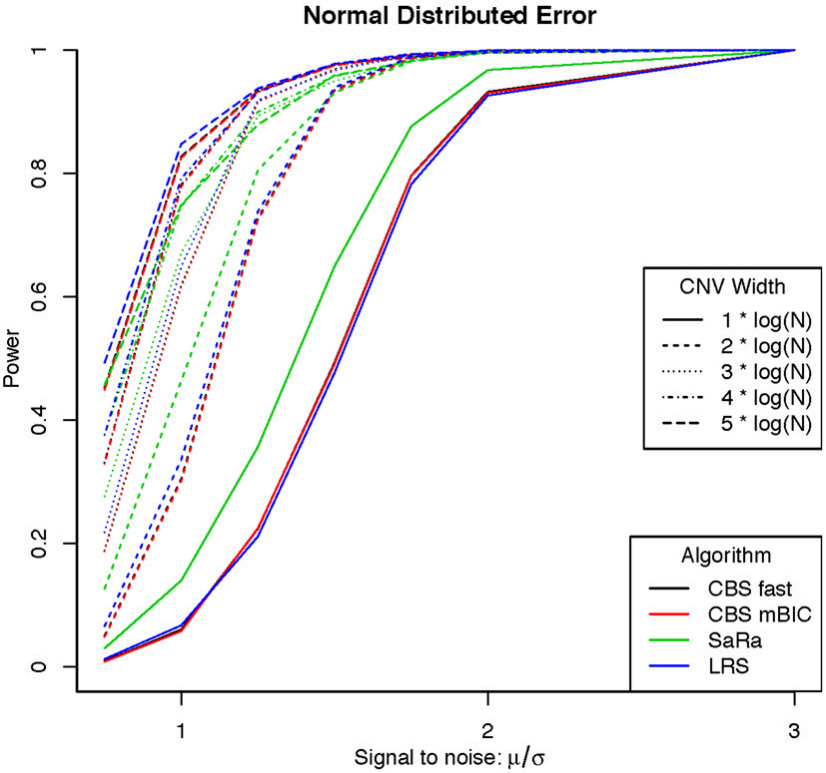
**Power for different signal to noise and CNV width under a normal distribution**.

**Figure 2:**
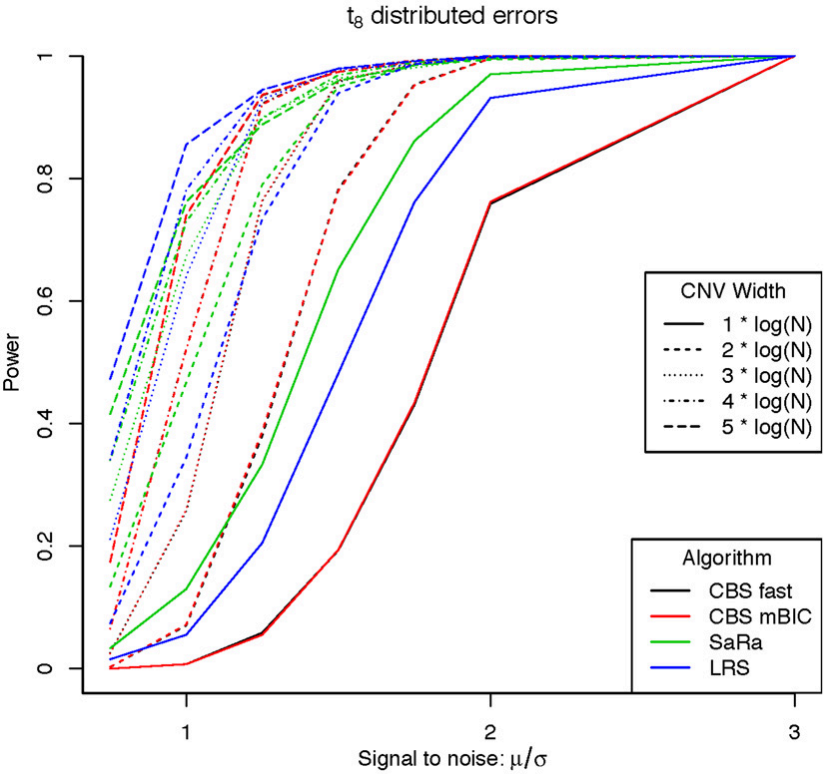
**Power for different signal to noise and CNV width under a heavy tailed (t_8_) distribution**.

An aberration was considered detected if a break point is found within 8 probes for both break points. As expected the power was not affected by actual genome size because aberration width was increased at the appropriate rate. Figures 1, 2 display the results for each method, aberration width, and signal to noise for both error distributions averaged over the different genome lengths. It is clear that the sparse signal methods are substantially more powerful than CBS (*p* < 0.001) and the difference in power is even more dramatic for the t-error distribution than for the normal distribution.

Interestingly, Sara appears to outperform the other algorithms for the smallest 3 CNV widths, but the power stops increasing for larger width aberrations. This pattern directly corresponds to the fact that the smallest 3 CNV widths corresponded exactly to the Sara window lengths used. This highlights how power to detect CNV with an arbitrary aberration width will be similar to the power to detect an aberration of size equal to the nearest Sara window size.

For the LRS algorithm, we have a similar pattern in that the power will be maximized for aberrations with width equal to L. Aberrations larger than L will be broken into multiple aberrations that must be joined after segmentation or a single region that will be a sub part of a larger region. The first situation can be easily handled because the multiple aberrations will be non-zero and they can be found quickly. The second situation is best handled by a global method such as CBS.

The Fused Lasso has a strange power curve for both error distributions. This is mostly likely due to how the smoothing parameters are selected in the software. As better and more flexible software (i.e., allow users to choose tuning parameters) becomes available, it would be interesting to implement this method across many settings. In this case, it is the worst performing algorithm for power.

### False positives

Table 1 shows the average number of false positives for each algorithm and error distribution. This table indicates that the permutation approach for CBS maintains robustness to noise. CBS interestingly has fewer false positives for t-distribution error, but this is likely explained by the substantial decrease in power. We once again see that the Fused Lasso has sub par performance with the highest number of false positives in the normal distribution and it has a higher number than CBS for the heavy tailed errors. Due to the poor performance of the Fused Lasso here and in other work (Niu and Zhang, 2012) we do not use it for the real data evaluation.

**Table 1:** **Average number of false positive break points by error structure for simulation**.

	**Normal**	***t*_8_**
CBS	0.430	0.287
CBS-BIC	0.372	0.264
SaRa	0.617	1.401
LRS	0.324	4.424
Fused Lasso	0.805	1.060

Max S.E. (paired) = 0.12.

**Figure 3:**
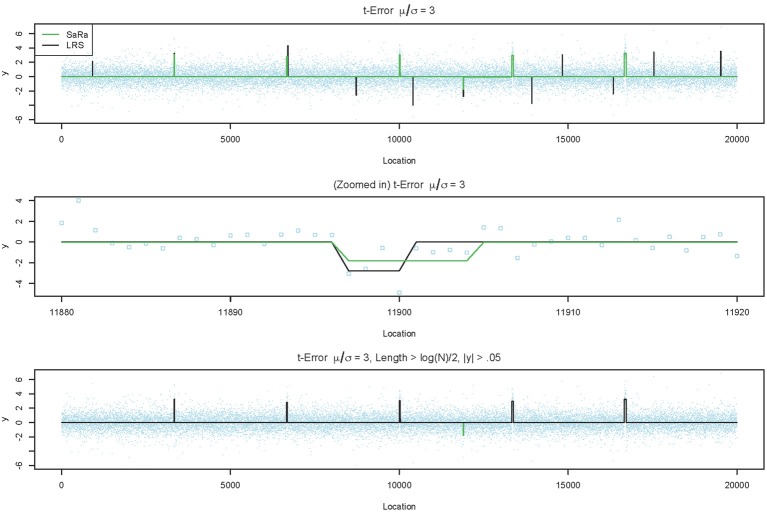
**LRS and SaRa results on simulation before and after pruning**. The top figure runs the standard LRS and SaRa procedure and results in a few false positives. The middle figure shows the difference width and magnitude between two algorithms for one false positive. The bottom figure is the result of removing small width aberrations. This now matches the true simulated profile for LRS but the false positive for SaRa remains.

Both LRS and Sara appear to have unacceptably high false positive rates for heavy tailed distributions. However, we provide a representative example in Figure 3, to demonstrate that the both algorithms tends to have false positives as regions with very small widths and these are extremely easy to remove. However, we also highlight in Figure 3, that the false positive for the SaRa algorithm has wider widths for the same false positive and so a larger threshold is required to remove it. The variable window size of the LRS scan statistic adjusts differently to the data than the fixed window of the Sara scan. The Sara algorithm also requires an additional magnitude threshold step because this algorithm does not call set regions as 0. Also, we do not show data, but the number of false positives does increases with genome length when errors are heavy tailed.

The simple pattern of false positives along with the increased power suggest both the Sara and LRS algorithm could be used to provide better concordance between technical replicates as compared to other more global algorithms. One would have to make small adjustments to remove small width aberrations, but such adjustments are standard practice currently (Pinto et al., 2011).

### Real data

**Figure 4:**
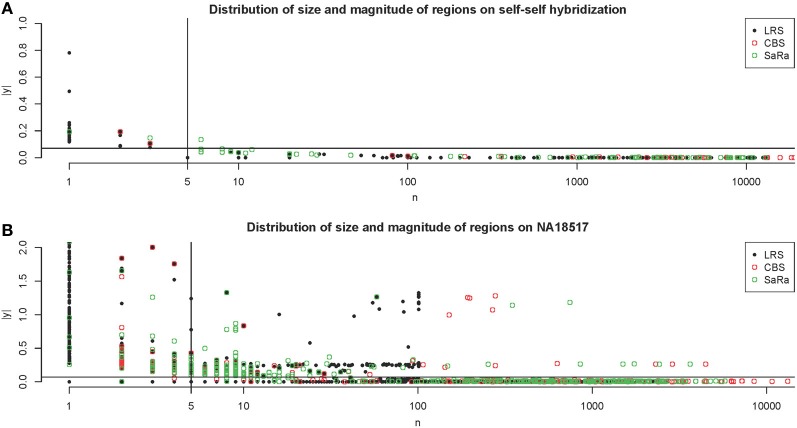
**Distribution of regions on Self-Self Hybridization and sample NA18517**. **(A)** shows the distribution of regions for each method on one technical replicate of the self-self hybridization. The thresholds used here are 5 probes and 1.5 times the median absolute deviation. **(B)** shows the same figure for the non self-self hybridization. The differences between the algorithms are more apparent and it is clear how the threshold rules may affect the calls for different algorithms differently.

The NA10851 data shows that there are a large number of false positives in the technical replicates but most can be easily removed. This gives us a good basis for the amount of segmentation induced by platform effects. We can see many results similar to our simulation. CBS has large number false positives and the sparse signal methods tend to have even more false positives. We can see in Figure 4A, that the LRS algorithm once again tends to have large magnitude calls have widths less than 5 probes while the SaRa algorithm tends to have slightly larger widths with smaller magnitudes. If we use a standard threshold of 5 probes (Pinto et al., 2011) and 1.5 times the median absolute deviation, we can remove nearly all calls for this repetition. We use these thresholds to post-process calls for the rest of the samples.

**Figure 5:**
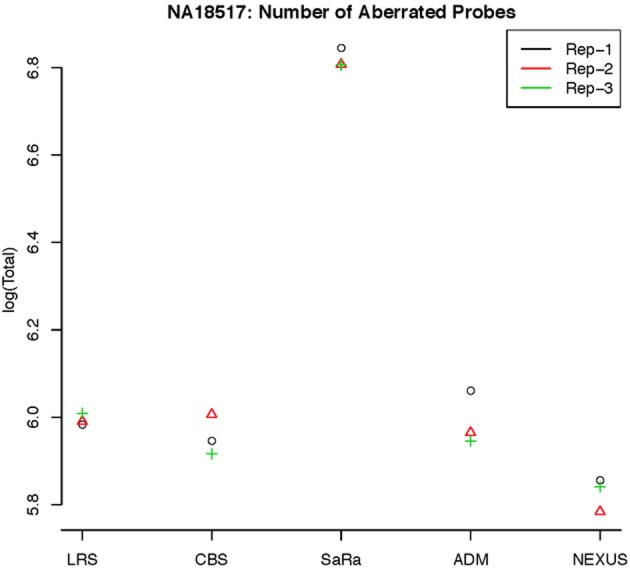
**log(Number) of unique CNV identified by each algorithm for HapMap sample NA18517 for each technical replicate**.

**Figure 6:**
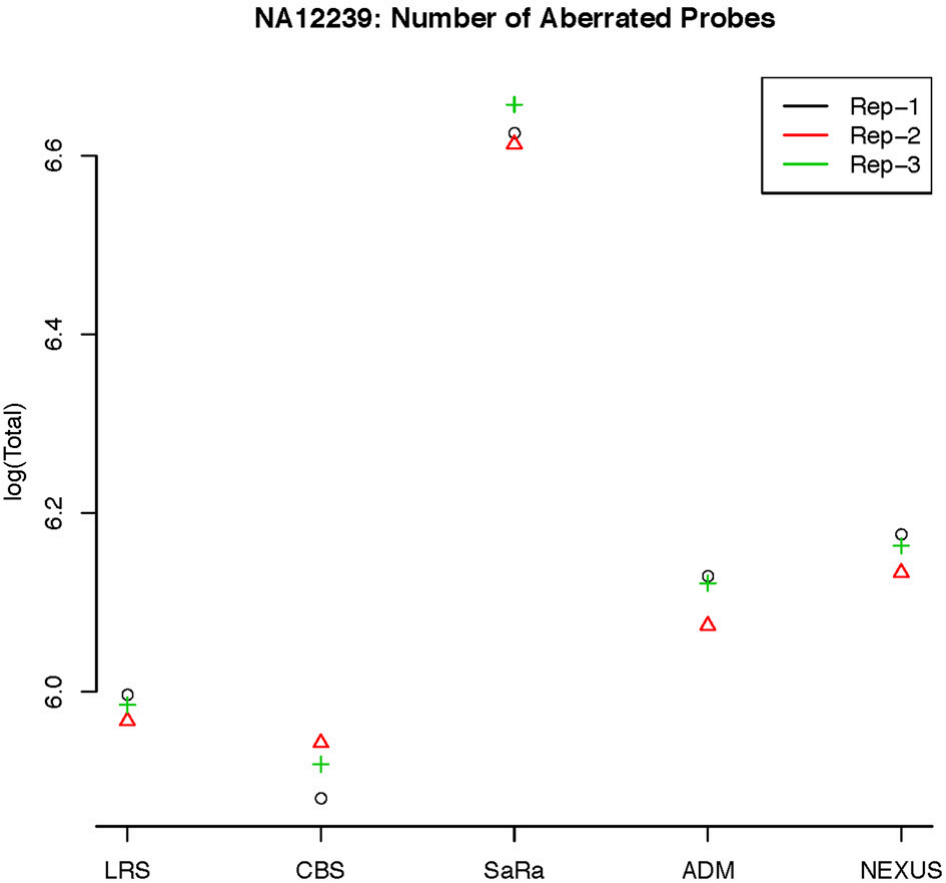
**log(Number) of unique CNV identified by each algorithm for HapMap sample NA12239 for each technical replicate**.

This contrast between methods becomes more interesting as we focus on the non self-self hybridizations. In Figure 4B, it is clear that the same simple thresholds will results in substantially more calls for SaRa than LRS. While LRS once again has many low width calls, the SaRa algorithm has more variability. We also see that there are many regions detected by CBS and SaRa that are larger than the scan width of 100 chosen for LRS. This suggests the need to use a global algorithm in conjunction with LRS to obtain accurate break point detection for larger regions. Both samples also have same consistent pattern in terms of number of probes called by an algorithm for both of the samples (Figures 5, 6).

**Figure 7:**
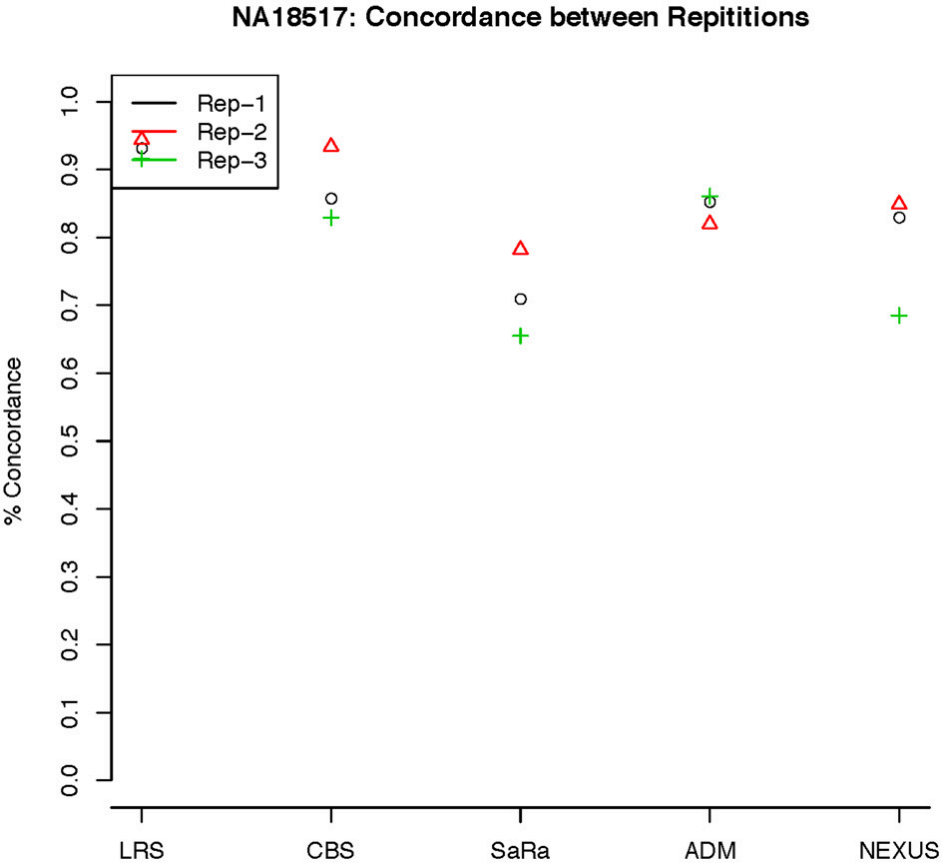
**This is % Concordance between repetitions for the same method for NA18157**. Each sample was measured in triplicate so the probes declared CNV from one method on a repetition are compared to the probes declared as a CNV from the same method on a different repetition.

**Figure 8:**
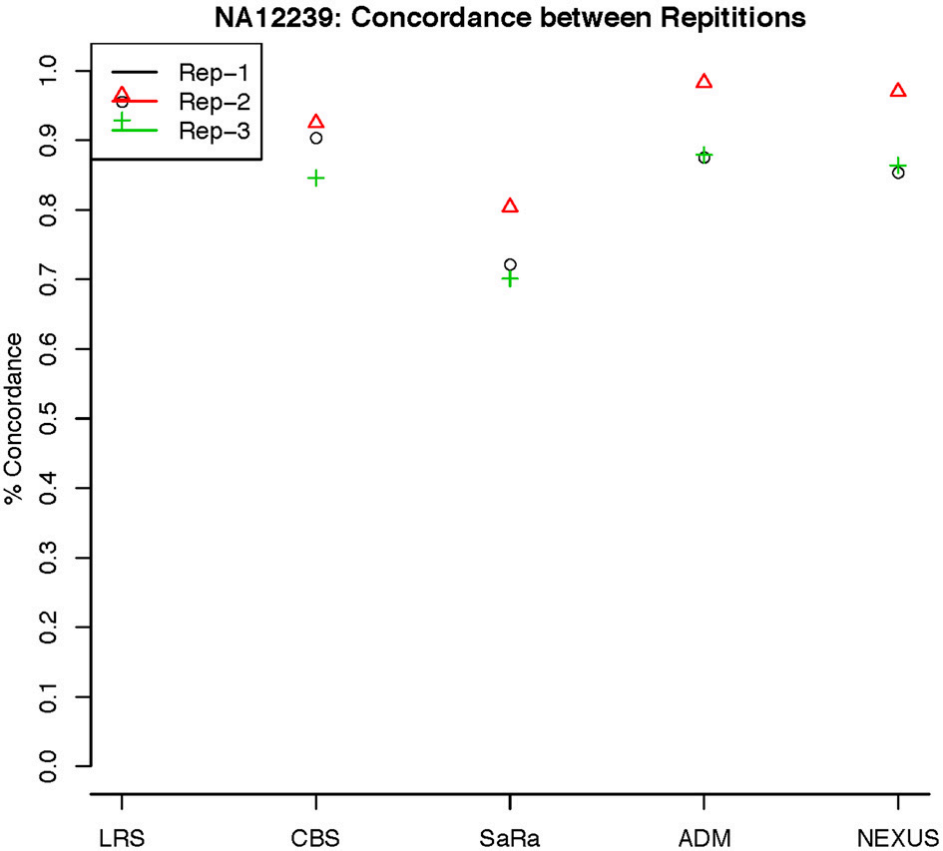
**This is % Concordance between repetitions for the same method for NA12239**. Each sample was measured in triplicate so the probes declared CNV from one method on a repetition are compared to the probes declared as a CNV from the same method on a different repetition.

To objectively contrasts algorithms, we define the percent concordance between two methods/replicates as the total number of probes called as a CNV by both methods divided by the geometric mean of the number or probes called as CNV by either method. This value gets reduced dramatically for methods like SaRa that call large numbers of probes. The lower concordance across replicates compared to other methods, seen in Figures 7, 8 for NA181517 and 10 for NA12239, indicates that SaRa is calling large amounts of probes that are not as easily reproducible.

**Figure 9:**
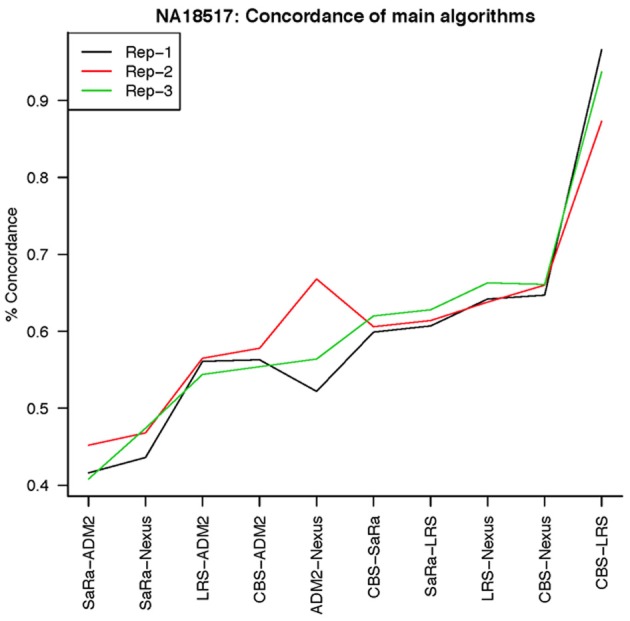
**This is % Concordance between methods for HapMap sample NA18517**. Percent concordance is defined as the number of probes called as a CNV on two algorithms divided by the geometric mean of the total number of probes called significant by the two algorithms.

**Figure 10:**
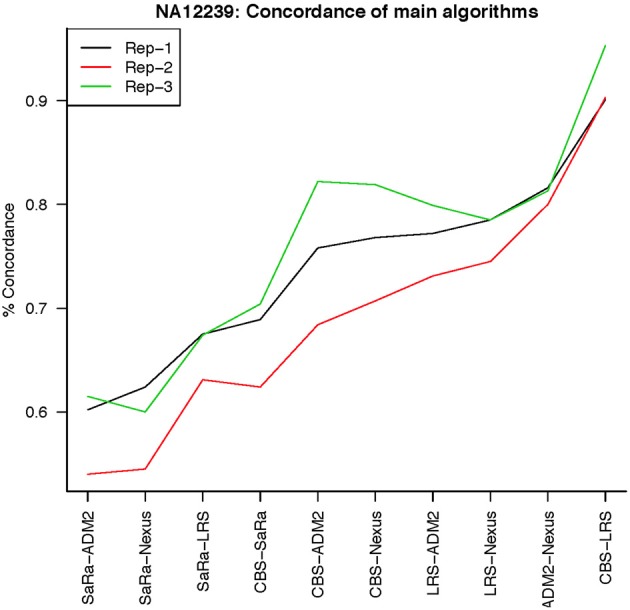
**This is % Concordance between methods for HapMap sample NA12239**. Percent concordance is defined as the number of probes called as a CNV on two algorithms divided by the geometric mean of the total number of probes called significant by the two algorithms.

The remarkable result here is the similarity between LRS and CBS both with each other and across replicates. Without processing the LRS algorithm detects nearly 4–5 fold more false positives. After we threshold the calls, we see that the LRS has over 90% concordance with the CBS algorithm and with the LRS results on other replicates (Figures 7–10). This is nearly a 50% increase relative to other combinations in particular the ADM2-Nexus combination and it is higher than previous results reported in literature (Pinto et al., 2011). Similar to simulations, we also see that the LRS calls a few more probes significant than CBS, and the similarity across replicates suggests that these are reproducible.

## Discussion

In this paper, we compared and assessed the usefulness of two new calling algorithms relative to popular standard methods. It is clear that these methods have substantially higher power to detect CNV, but they are less robust to assumptions especially deviations from normality. However, we also find that it is easy to understand how heavy tails affect these algorithms and thus it is easy to remove these effects.

In the real data, we found that the LRS and CBS methods have a concordance nearly 50% higher than previous methods after using thresholds for clear false positives. Standard methods like ADM and Nexus do not achieve the same levels of similarly. Since the usual practice is to use multiple algorithms along with basic thresholds, our recommendation would be to first use CBS to find the larger calls because it is more robust to heavy tails. This should then be augmented with the LRS procedure with some pruning to evaluate specific regions. It should be noted that the results and conclusions in both simulations and real data could be limited to our current implementation of the software. Better implementation along with better methods (i.e., choice of window for SaRa) could lead to different results and conclusions.

Future work would use calls from the 10 other platforms to try and get a better sense of the false positive and false negative rates of various discrepancies. As sequencing technologies become more common, it would be useful to obtain break point locations using deep sequencing that could then be used to more accurately assess the array technologies. Also, evaluation of these same HapMap samples on sequencing platforms would allow for all major CNV platforms to be compared thoroughly. This is important because sequencing platforms tend to create additional problems both computationally due to size of data and methodology due to different assumptions being required (Duan et al., 2013). Methods used must have lower computational complexity as well as be more robust. An even larger problem with sequencing technologies is that the biases present in data are less understood.

Overall, in this work, we saw clear differences in the methods that were utilized and could easily make conclusions. However, employing statistical models to CNV platform comparison is still currently not done and it would be a useful tool for the community, as technologies get higher in resolution. Until, problems with sequencing technologies are effective reduced, array based technology will continue to be a popular resource for study of CNV. We hope that this work will be useful to others in choosing the appropriate method and platform for their study.

### Conflict of interest statement

The authors declare that the research was conducted in the absence of any commercial or financial relationships that could be construed as a potential conflict of interest.
